# Cost-effectiveness analysis of Tislelizumab vs Sorafenib as the first-line treatment of unresectable hepatocellular carcinoma

**DOI:** 10.1371/journal.pone.0295090

**Published:** 2024-03-04

**Authors:** Qiuping Chen, Quan Sun, Jing Zhang, Baixue Li, Quansheng Feng, Jibin Liu

**Affiliations:** 1 College of Clinical Medicine, Chengdu University of Traditional Chinese Medicine, Chengdu, China; 2 Guangzhou ZhongWei Public Health Technology Accessment Institute, Guangzhou, China; Kaohsiung Medical University Hospital, TAIWAN

## Abstract

**Background:**

To evaluate the cost-effectiveness of Tislelizumab vs Sorafenib as the first-line treatment of unresectable hepatocellular carcinoma (HCC) from the perspective of the Chinese health service system.

**Methods:**

A lifetime partitioned survival model (PSM) was developed to cost-effectively analyze Tislelizumab vs Sorafenib as the first-line treatment of unresectable HCC. The clinical and safety data were derived from a recently randomized clinical trial (RATIONALE-301). Utilities were collected from the published literature. Costs were obtained from an open-access database (http://www.yaozh.com) and previous studies. The model cycle was 21 days, according to the RATIONALE-301 study, and the simulation period was patients’ lifetime. Long-term direct medical costs and quality-adjusted life-years (QALYs) were determined. The incremental cost-effectiveness ratio (ICER) was used as the evaluation index. one-way sensitivity analysis (OSWA) and probabilistic sensitivity analysis (PSA) were used to analyze the uncertainty of parameters and to adjust and verify the stability of the baseline results.

**Results:**

The Tislelizumab group generated a cost of $39,746.34 and brought health benefits to 2.146 QALYs, while the cost and utility of the Sorafenib group were $26750.95 and 1.578 QALYs, respectively. The Tislelizumab group increased QALYs by 0.568, the incremental cost was $12995.39, and the ICER was $22869.64/QALY, lower than the willingness to pay threshold (WTP). OSWA results showed that the utility of progressed disease (PD), cost of Camrelizumab, and cost of Tislelizumab were the main factors affecting the ICER. PSA results showed that, within 1000 times the Monte Carlo simulation, the cost of the Tislelizumab group was lower than three times the per capita gross domestic product (GDP) of China ($37653/QALY). The cost-effectiveness acceptability curves (CEAC) revealed that when WTP was no less than $12251.00, the Tislelizumab group was the dominant scheme, and the economic advantage grew with an increasing WTP. When WTP ≥ $19000.00, the Tislelizumab group became the absolute economic advantage.

**Conclusion:**

Under the current economic conditions in China, the Tislelizumab therapeutic scheme is more cost-effective than the Sorafenib therapeutic scheme for treating patients with unresectable HCC.

## Introduction

Primary hepatoma is a malignant tumor of the digestive system and the sixth most common malignancy worldwide [1]. As a significant type of primary hepatoma, hepatocellular carcinoma (HCC) is the second leading cause of cancer-related deaths globally [2] and the fourth leading cause of cancer deaths in China [3]. On the one hand, most HCC patients already have advanced disease at diagnosis [4] and cannot be treated with transplantation, ablation, surgical resection, and radiotherapy [5,6]. On the other hand, the efficacy of tyrosine kinase inhibitors (TKI) as first-line chemotherapy regimens for HCC is minimal [7]. The above two factors lead to the 5-year survival rate remains poor [8].

In recent years, Studies have increasingly shown that programmed death-1 (PD‐1) and programmed death ligand-1(PD-L1) are the most prevalent immune checkpoint inhibitors, which have shown some clinical benefit in late-stage or metastatic HCC, such as Nivolumab, Pembrolizumab and Sintilimab. In the CheckMate 040 study, Nivolumab plus Ipilimumab, compared with Nivolumab, improved efficacy and safety in patients with advanced HCC previously treated with Sorafenib [9]. A recent randomized controlled study (ORIENT-32) indicated that Sintilimab plus a Bevacizumab biosimilar had improved progression-free survival (PFS) and overall survival (OS) in patients with unresectable hepatocellular carcinoma [10]. Pembrolizumab demonstrated antitumor activity and safety in the phase II KEYNOTE-224 trial in previously treated patients with advanced HCC [11].

Recently, RATIONALE-301 research evaluated for the first time the efficacy and safety of Tislelizumab vs Sorafenib as a first-line Treatment for Unresectable HCC [12]. The results showed that the OS of the Tislelizumab and Sorafenib groups was 15.9 months and 14.1 months, respectively. Patients in the Tislelizumab group also had a higher objective response rate (14.3% vs 5.4%). From a security perspective, the incidence of treatment-related adverse events (TRAE) in Tislelizumab ≥3 was lower (22.2% vs. 53.4%) compared with Sorafenib. In short, it can improve patients’ OS, PFS, and Objective Response Rate (ORR) and is a safe and effective treatment. At present, there is no economic research on Tislelizumab as the first-line treatment of advanced HCC in China. Therefore, from the perspective of the Chinese healthcare system, this study explores the cost-effectiveness of Tislelizumab as the first-line treatment option in patients with advanced HCC by establishing a partitioned survival model (PSM) to provide clinicians with more options.

## Methods

### Data source and the study population

The patient data and treatment plan information used in this study were derived from the RATIONALE-301 study. The target population was consistent with the target patient population of the RATIONALE-301 trial. Inclusion criteria: age 18–75 years; has received no prior systemic therapy for HCC; at least one measurable lesion in the liver according to the mRECIST criteria; ECOG PS score ≤ 1; Child-Pugh class A; normal liver functions. Exclusion criteria: has known fibrolamellar HCC, sarcomatoid HCC, or mixed cholangiocarcinoma and HCC histology; has tumor thrombus involving main trunk of portal vein or inferior vena cava; has received within 28 days before randomization loco-regional therapy and any prior immunotherapy to the liver; any active immune deficiency or autoimmune disease and/or has a history of any immune deficiency or autoimmune disease that may relapse. A total of 662 patients were included, patients were randomized to Tislelizumab (338), 200 mg intravenously every 3 weeks; or Sorafenib (324), 400 mg orally twice daily, until symptomatic deterioration associated with disease progression. unacceptable toxic effects, or study withdrawal. Camrelizumab in combination with FOLFOX4 regimen was selected as a second-line treatment for all patients with progressive disease in accordance with the CSCO Guidelines for the Diagnosis and Treatment of Primary Liver Cancer (2022).

### Model construction

According to the disease progression of hepatoma, a three-state PSM of PFS, progressed disease (PD), and death was established for cost-effectiveness analysis. It was assumed that all patients entered the model from the PFS state, the model cycle was set to 21 days, and the simulation time was lifetime horizon. According to the “Chinese Pharmacoeconomic Evaluation Guidelines (2020)”, the discount rate of both cost and utility is set to 5%. The main outputs of the model were total costs, quality-adjusted life years (QALYs), and incremental cost-effectiveness ratio (ICER). The survival curve fitting of this study was painted using R 4.2.0 software, and the PSM model was constructed with Microsoft Excel 2019 software.

### Survival and progression risk estimation

**Fig 1:**
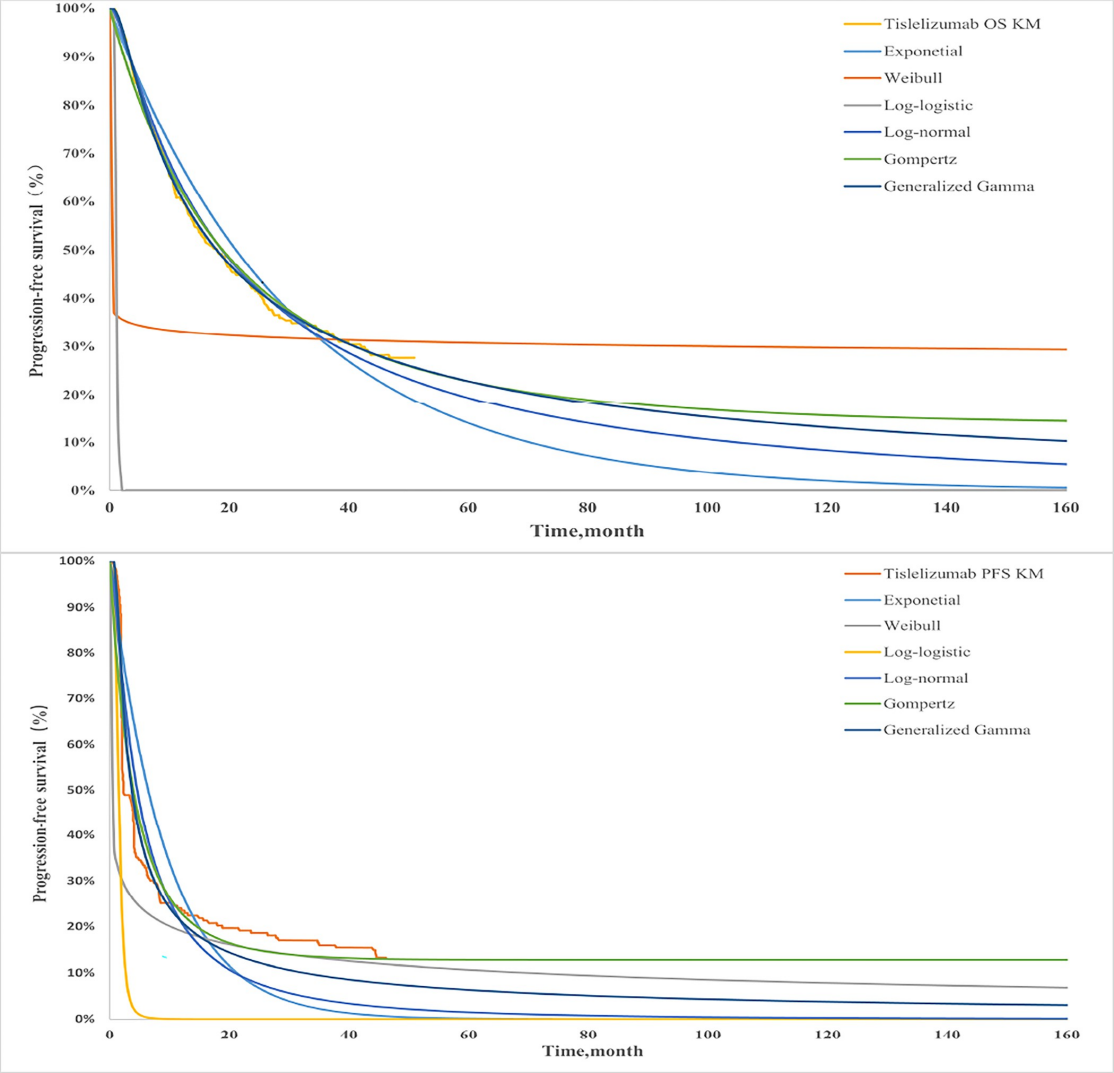
Results of the survival curve fit the Tislelizumab of the base-case analysis in the partitioned survival model.

**Fig 2:**
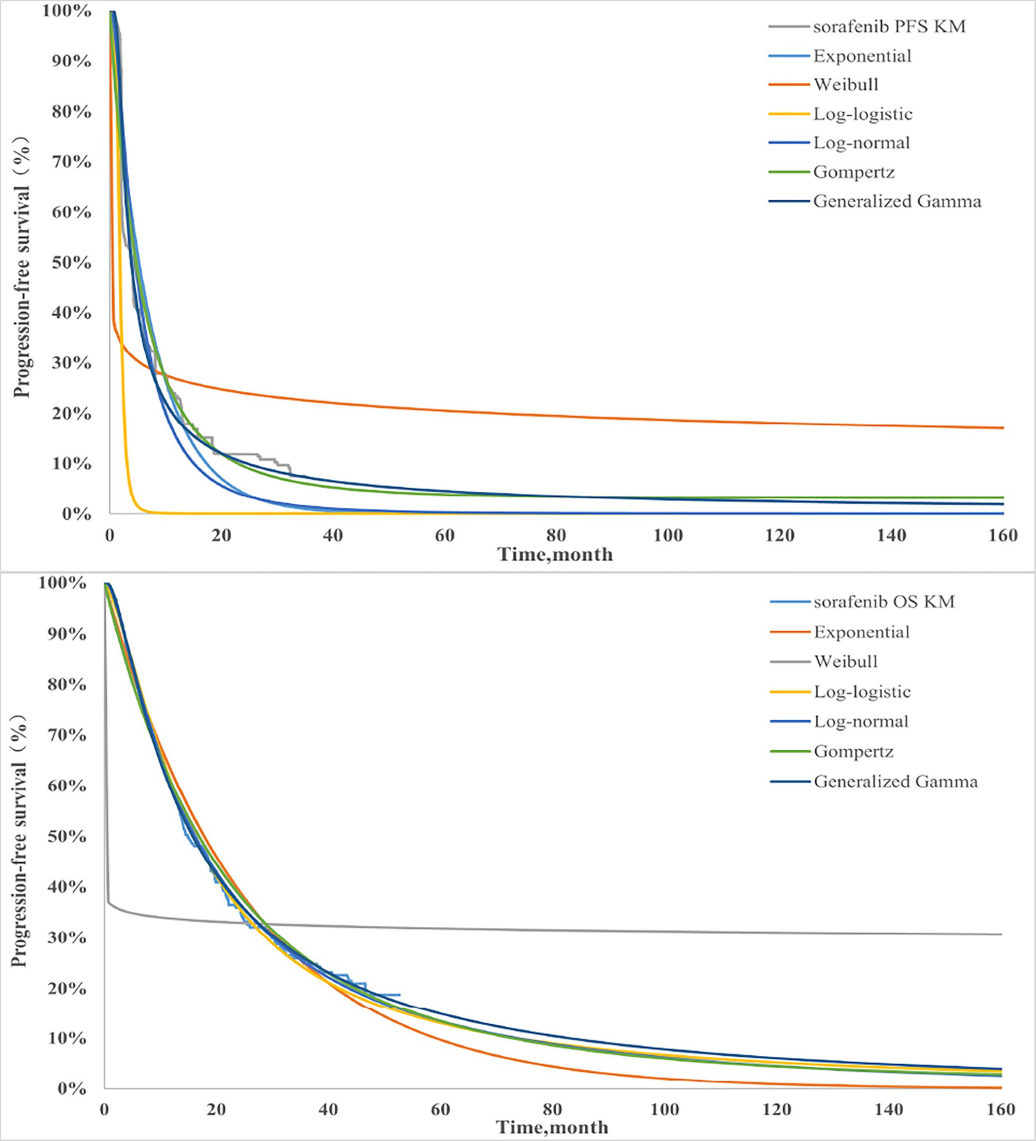
Results of the survival curve fit the Sorafenib of the base-case analysis in the partitioned survival model.

We used the Getdata Graph Digitizer software to extract the survival data of the RATIONALE-301 trial and then reconstructed the pseudo-individual patient data by using the survHE and survival package of R 4.2.0 software. Log-normal, Log-logistic, Weibull, Exponential, Gen.Gamma and Gompertz distributions were selected to calculate the parameters as previously described (Figs 1 and 2). Based on the Akaike information criterion (AIC) and Bayesian information criterion (BIC), the best-fitting parametric distribution curves of both PFS and OS of the two groups was selected (Table 1).

**Table 1:** Model parameters: Baseline values, ranges, and distributions for sensitivity analysis.

Variable	Baseline value ($)	Lower	Upper	Distribution	Source
**Generalized Gamma OS survival model in the Tislelizumab group**	mu = 2.569464; sigma = 1.402937; Q = -0.645479	ND	ND	ND	Model fifitting
**Log-normal OS survival model in the Sorafenib group**	Shape = 2.78300; scale = 1.18198	ND	ND	ND	Model fifitting
**Generalized Gamma PFS survival model in the Tislelizumab group**	mu = 0.74281; sigma = 0.76369; Q = -1.74938	ND	ND	ND	Model fifitting
**Generalized Gamma PFS survival model in the Sorafenib group**	mu = 0.887573; sigma = 0.671562; Q = -1.684204	ND	ND	ND	Model fifitting
**Cost of drugs**					
**Tislelizumab**	192.85	154.28	231.42	gamma	http://db.yaozh.com
**Sorafenib**	3.19	2.55	3.83	gamma	http://db.yaozh.com
**Camrelizumab**	360.73	288.58	432.88	gamma	http://db.yaozh.com
**Oxaliplatin**	47.13	37.70	56.56	gamma	http://db.yaozh.com
**Calcium Folinate**	13.72	10.98	16.46	gamma	http://db.yaozh.com
**5-fluorouridine**	4.06	3.25	4.87	gamma	http://db.yaozh.com
**Cost of follow-up**	84.77	67.82	101.72	gamma	[13]
**Cost of AEs**					
**Aspartate Aminotransferase (AST) level increased**	59	47.20	70.80	gamma	[14]
**Alanine Aminotransferase (ALT) level increased**	59	47.20	70.80	gamma	[14]
**Blood bilirubin level increased**	124.90	99.92	149.88	gamma	[15]
**Rash/Pruritus**	7	5.60	8.40	gamma	[14]
**Platelet count decreased**	332.15	265.72	398.58	gamma	[16]
**Fatigue**	3	2.40	3.60	gamma	[14]
**Diarrhea**	88.38	70.70	106.06	gamma	[17]
**Decreased appetite**	107.38	85.90	128.86		[18]
**Weight decreased**	75.20	60.16	90.24	gamma	[15]
**Hypertension**	64.01	51.21	76.81	gamma	[17]
**Alopecia**	183	146.40	219.60	gamma	[19]
**Palmar-plantar erythrodysesthesia syndrome**	145.65	116.52	174.78	gamma	[17]
**Risk of AEs**					
**Tislelizumab group**					
**AST level increased**	23.1%	20.79%	25.41%	beta	[12]
**ALT level increased**	16.6%	14.94%	18.26%	beta	[12]
**Blood bilirubin level increased**	12.4%	11.16%	13.64%	beta	[12]
**Rash/Pruritus**	20.5%	18.45%	22.55%	beta	[12]
**Platelet count decreased**	7.1%	6.39%	7.81%	beta	[12]
**Fatigue**	6.2%	5.58%	6.82%	beta	[12]
**Diarrhea**	5.6%	5.04%	6.16%	beta	[12]
**Decreased appetite**	5.0%	4.50%	5.50%	beta	[12]
**Weight decreased**	3.3%	2.97%	3.63%	beta	[12]
**Hypertension**	2.7%	2.43%	2.97%	beta	[12]
**Alopecia**	0.3%	0.27%	0.33%	beta	[12]
**Palmar-plantar erythrodysesthesia syndrome**	0.3%	0.27%	0.33%	beta	[12]
**Sorafenib group**					
**AST level increased**	28.7%	25.83%	31.57%	beta	[12]
**ALT level increased**	25.0%	22.50%	27.50%	beta	[12]
**Blood bilirubin level increased**	20.7%	18.63%	22.77%	beta	[12]
**Rash/Pruritus**	21.6%	19.44%	23.76%	beta	[12]
**Platelet count decreased**	15.1%	13.59%	16.61%	beta	[12]
**Fatigue**	10.5%	9.45%	11.55%	beta	[12]
**Diarrhea**	39.2%	35.28%	43.12%	beta	[12]
**Decreased appetite**	12.0%	10.80%	13.20%	beta	[12]
**Weight decreased**	11.1%	9.99%	12.21%	beta	[12]
**Hypertension**	24.7%	22.23%	27.17%	beta	[12]
**Alopecia**	22.5%	20.25%	24.75%	beta	[12]
**Palmar-plantar erythrodysesthesia syndrome**	62.7%	56.43%	68.97%	beta	[12]
**Utility**					
**Utility of PFS (95CI)**	0.76	0.76	0.80	beta	[20]
**Utility of PD (95CI)**	0.68	0.60	0.68	beta	[20]

### Utility and cost

The utility values of PFS and PD status associated with advanced HCC were 0.76 and 0.68, respectively [20]. From the perspective of China’s healthcare system, only direct medical expenses were considered, including drug costs, follow-up costs, and treatment costs of adverse reactions (AEs) no less than grade 3 according to Chinese standard. The price of drugs was obtained from the latest market bid price in public databases, which were all up to date in 2023. It was assumed that the patient’s average weight and average body surface area (BSA) were 65 kg and 1.72 m^2^ [21], respectively. Follow-up costs were derived from references. The cost in this article is calculated in RMB. The incidence of AEs was obtained from the RATIONALE-301 trial. Because the RATIONALE-301 trial did not classify AEs, this study could not consider the management costs of serious adverse reactions. To simplify the model, only adverse events with an incidence rate of ≥ 10% were included, including AST level increased, ALT level increased, blood bilirubin level increased, rash/pruritus, platelet count decreased, fatigue, diarrhea, decreased appetite, weight decreased, hypertension, alopecia, palmar-plantar erythrodysesthesia syndrome [12]. The details are given in Table 1.

### Sensitivity analysis

We consider that the parameters included in the model may fluctuate within a certain range. The one-way sensitivity analysis (OWSA) was performed to test the robustness of the parameters in the model. In the OWSA, input parameters were adjusted one-by-one to their respective minimum and maximum values, with a range of the 95% confidence intervals reported in the referenced literature or a ± 20% change from the base-case value (the rate of AEs were set over a range of ± 10% of the baseline values), in order to ascertain the variables that significantly influenced the economic outcomes. The results are shown in the form of a cyclone diagram. Probabilistic sensitivity analysis (PSA) can evaluate the variation of multiple parameters and 1000 Monte Carlo simulations to obtain scatter plots and good curves to test the acceptable probability of different optimal strategies at different WTP thresholds.

### Scenario analysis

Considering the potential impact of discount on the results of pharmacoeconomics, and according to the requirements of the "China Pharmacoeconomics Evaluation Guide 2020", the discount rate was 0–8%. In this study, 3% and 8% were used as the discount rates in the model for scenario analysis to explore the economics of the two treatment options under different discount rates.

## Results

### Base-case analysis

The results of base-case analysis are shown in Table 2. The aggregate costs of the Tislelizumab group was $39746.34, and the cumulative health benefit gained was 2.146 QALYs; The aggregate cost and cumulative effect of the Sorafenib group were $26750.95 and 1.578 QALYs, respectively. The incremental cost of the Tislelizumab group was $12995.39, and the incremental effect was 0.568 QALYs. The ICER was $22869.64/QALYs when using 3 times per capita GDP of China in 2022 as the criterion for the willingness to pay (WTP) threshold. The ICER was lower than the WTP threshold.

**Table 2:** Base-case analysis results.

Group	Cost/$	Utility (QALY)	Incremental cost ($)	Incremental utility (QALY)	ICER ($/QALY)
**Tislelizumab group**	39,746.34	2.146	12,995.39	0.568	22,869.64
**Sorafenib group**	26,750.95	1.578	—	—	—

### Sensitivity analysis

#### One-way sensitivity analysis

**Fig 3:**
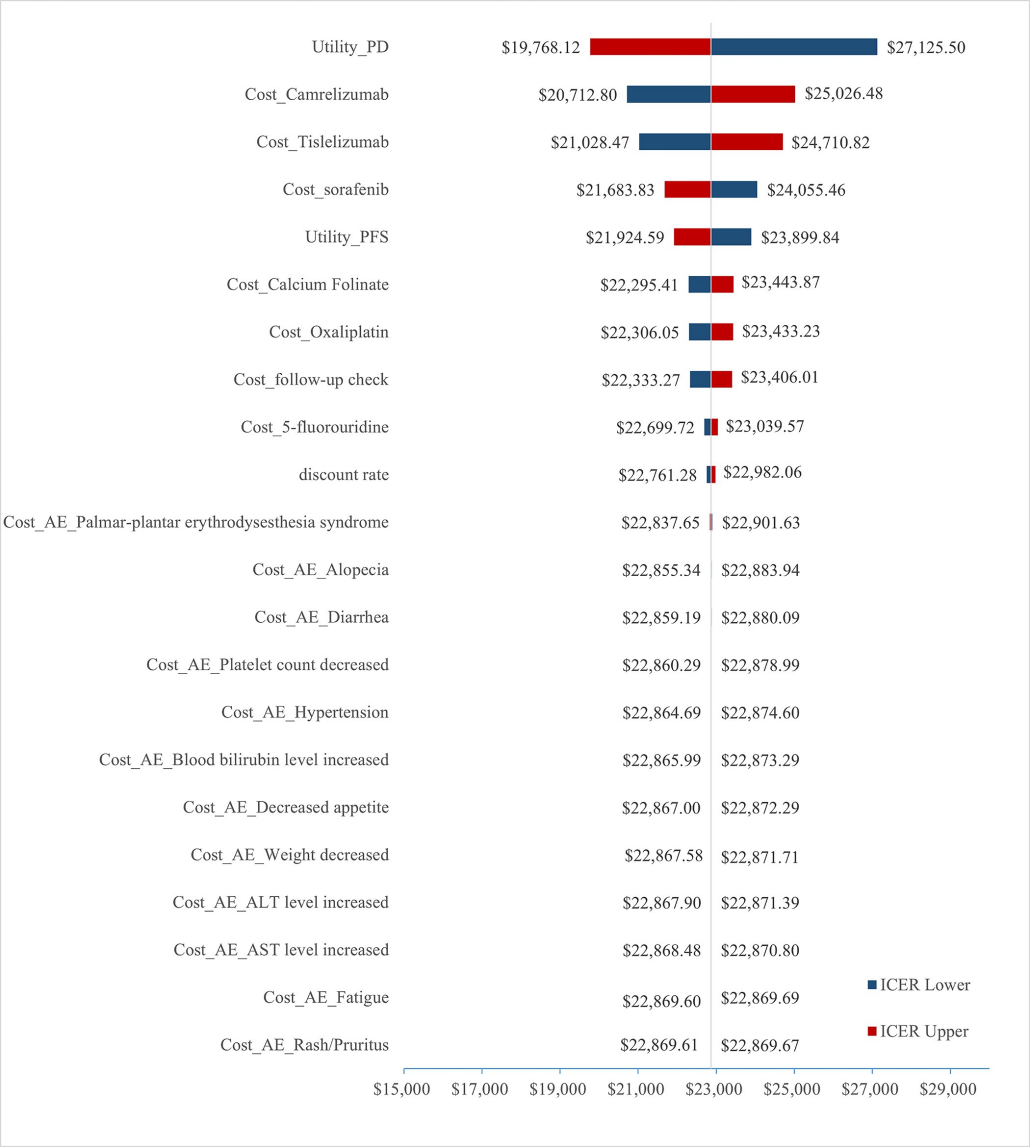
The one-way sensitivity analysis results presented by tornado diagram.

The results of the OWSA are shown in Fig 3, utility of PD, cost of Camrelizumab and cost of Tislelizumab were the primary factors that had a major impact on the results. The incidence of AEs and treatment costs of AEs had little effect on ICER. All parameters fluctuated within a limited range. The ICER value of the model results did not exceed 3 times the GDP per capita with a change in all uncertain parameters, and the results were consistent with the basic analysis results.

#### Probabilistic sensitivity analysis

**Fig 4:**
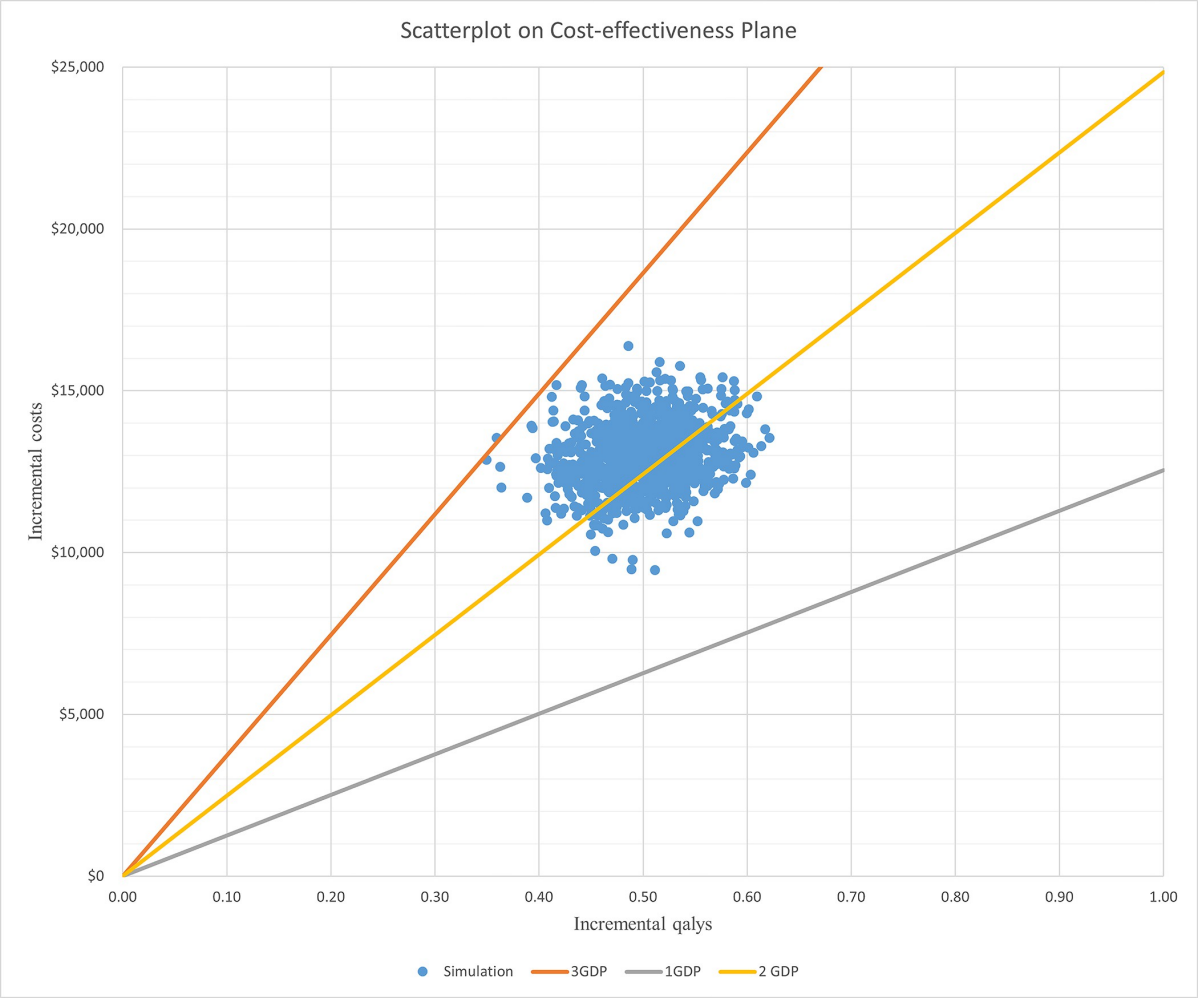
Incremental cost-effectiveness scatter plot.

**Fig 5:**
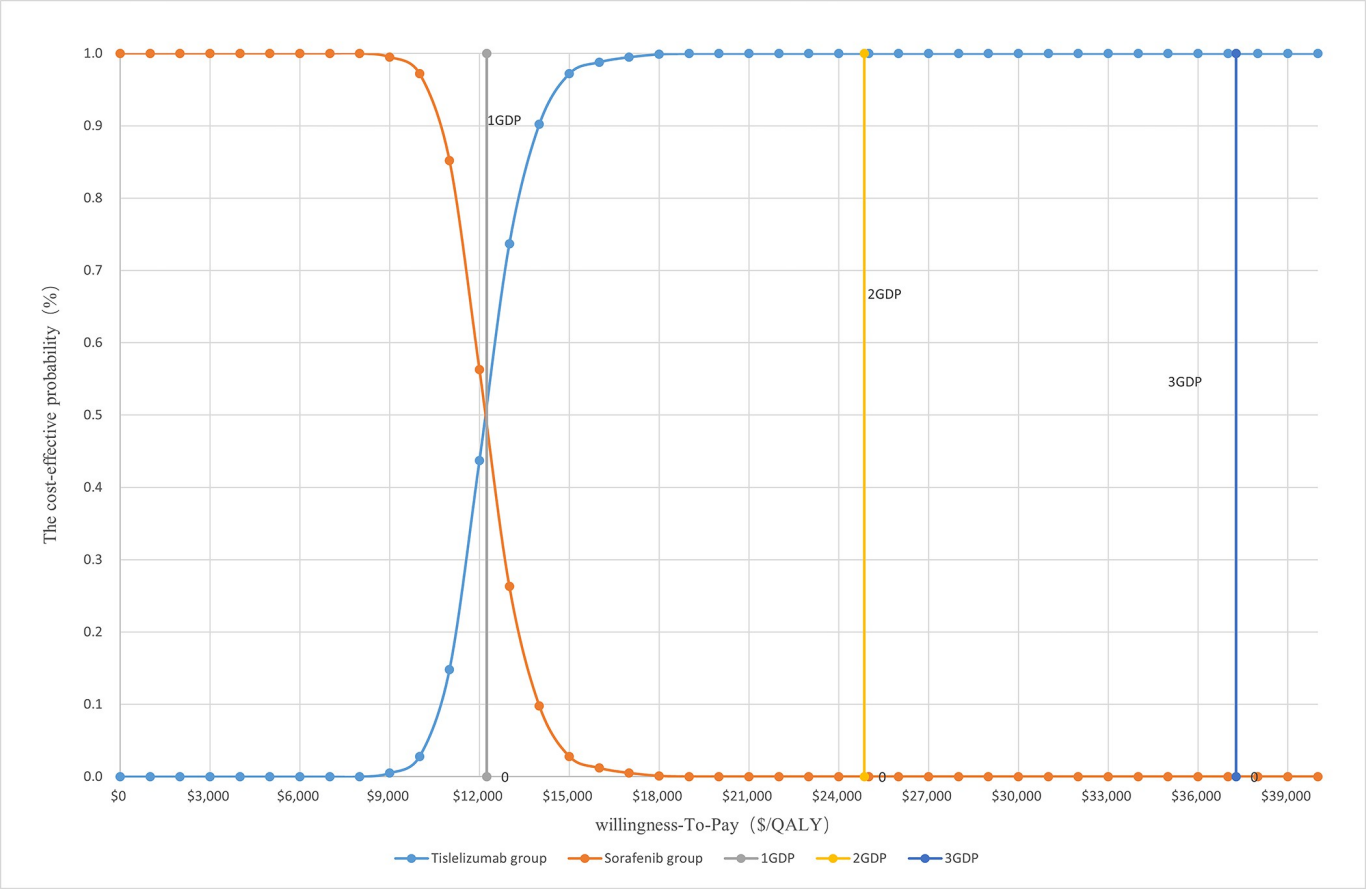
Cost-effectiveness acceptability curve.

As shown in Fig 4, if the WTP is equal to 1 time the GDP per capita, all the scattered points are above the WTP line, and the Tislelizumab group is not economically efficient. When WTP is equal to 3 times the GDP per capita, the Tislelizumab group is more economical than the Sorafenib group. The CEAC (Fig 5) shows that the economical effectiveness of Tislelizumab as the first-line treatment of HCC increased with an increase in the WTP. When WTP < $12251.00, the Sorafenib group was more economical. If WTP ≥ $19000.00, the economic probability of Tislelizumab was 100%. The CEAC shows that the economical effectiveness of Tislelizumab as the first-line treatment of HCC increased with a growing WTP. When WTP < $12251.00, the Sorafenib group was more economical. If WTP ≥ $19000.00, the probability that Tislelizumab was economical was 100%.

### Scenario analysis

If the discount rate was adjusted to 3% and 8%, ICER was $22,657.06/QALY and $23,218.77/QALY, respectively. Under the condition that the WTP threshold is 3 China’s GDP per capita in 2022, the Tislelizumab scheme is more cost-effective.

## Discussion

HCC is a common invasive malignant tumor that lacks effective treatment options, leading to poor survival and prognosis [22]. In recent years, the application of new drugs, such as immunomodulatory drugs, has significantly improved the treatment efficacy and prolonged survival of HCC patients [23]. Tislelizumab is a fully humanized monoclonal antibody against the PD-1 receptor, which blocks the interaction between PD-1 and its ligand, PD-L1, which unleashes the anti-tumor immune response [24]. Tislelizumab has achieved good efficacy and safety in the treatment of advanced or metastatic esophageal squamous cell carcinoma [25], recurrent or metastatic nasopharyngeal cancer [26], and advanced solid tumors [27]. The results of RATIONALE-301 research indicate that Tirelizumab monotherapy exhibits excellent efficacy, safety, and health-related quality of life [12]. Tirelizumab monotherapy enriches the choices for first-line treatment of advanced HCC and has been recommended by clinical guidelines both domestically and internationally. However, no relevant studies have evaluated the economics of Tislelizumab as a first-line treatment of unresectable HCC. Therefore, this is the first analysis by building a 3-state partitioned survival model to assess the efficacy and cost-effectiveness of Tislelizumab as the first-line therapy for patients with unresectable HCC from the perspective of the Chinese healthcare system, which may provide a reference for the formulation of related health insurance policies and clinical decision-making.

According to primary analysis results, the Tislelizumab group was associated with 0.568 more QALYs than the Sorafenib group, but the cost also increased by $12,995.39. The ICER was $22,869.64, which was lower than three times the per capita GDP of China in 2022. This study showed that Tislelizumab was a cost-effective first-line therapy for patients with unresectable HCC. The results of OWSA showed that the utility of PD, the cost of Camrelizumab and, the cost of Tislelizumab had a more significant impact on ICER. When the discount rate was adjusted to 3%, ICER was $22,657.06/QALY. If the discount rate was adjusted to 8%, ICER was $23,218.77/QALY. Under the condition that the WTP threshold is three times China’s GDP per capita in 2022, the Tislelizumab scheme is economical. Based on the above results, it can be concluded that the Tislelizumab regimen currently has a cost-effectiveness advantage over the Sorafenib regimen.

Although the model can simulate the natural development of the disease and evaluate the economics of the above two treatments for liver cancer under limited medical resources, there are some limitations. First, the survival curve fitting of this model was based on a large-scale phase III clinical trial with strict restrictions on patient conditions. Patients had explicit inclusion and exclusion criteria and high compliance. However, in clinical practice, disease regression and survival status are affected by many factors. Therefore, there may be differences between the various disease outcomes of patients in this study and those in the actual clinical situation. Second, there is a lack of utility values for patients with primary HCC in China. According to “the Guidelines for Pharmacoeconomic Evaluation 2020”, we obtained health utility values from published studies by conducting a literature search. Although the disease progression of patients with HCC was the same, the utility values may not precisely match the clinical reality of our patients due to ethnic differences, and the results of their analysis may be somewhat biased. Third, the treatment regimen of PFS in this study was centered around Tislelizumab and Sorafenib, while for PD, it revolved around Camrelizumab combined with FOLFOX4. In practical clinical practice, doctors can choose different treatment regimens depending on the patients’ individual situation.

## Conclusions

Under the current economic conditions in China, the Tislelizumab therapeutic scheme is more cost-effective than the Sorafenib therapeutic scheme for treating patients with unresectable HCC.
